# The first non-mammalian synapsid embryo from the Triassic of South Africa

**DOI:** 10.1371/journal.pone.0345016

**Published:** 2026-04-09

**Authors:** Julien Benoit, Vincent Fernandez, Jennifer Botha

**Affiliations:** 1 Evolutionary Studies Institute, University of the Witwatersrand, Johannesburg, South Africa; 2 European Synchrotron Radiation Facility, Grenoble, France; 3 GENUS: DSTI-NRF Centre of Excellence in Palaeosciences, University of the Witwatersrand, Johannesburg, South Africa; Museum für Naturkunde Berlin, GERMANY

## Abstract

Oviparity was likely the plesiomorphic reproductive condition for non-mammalian Synapsida, the stem-mammal group. Yet, despite nearly two centuries of research, no definitive fossil eggs of late Palaeozoic or early Mesozoic synapsids have been discovered. Here, three perinate specimens of the dicynodont genus *Lystrosaurus* from the Early Triassic of the South African Karoo Basin are examined using high-resolution CT and synchrotron scanning. One specimen, NMQR 3636, displays a tightly curled posture suggestive of an *in ovo* position and completely lacks tusks. Crucially, the lower jaw symphysis remains unfused—a developmental trait found only in pre-hatching embryos of modern birds and turtles. No calcified eggshell is preserved, so the egg might have been soft and leathery. The large size of the reconstructed egg suggests a precocial, non-milk-feeding developmental strategy. As a non-cynodont synapsid, *Lystrosaurus* offers a rare and valuable glimpse into reproductive biology far removed from the mammalian crown group. Unlike the more derived, mammal-like cynodont *Kayentatherium*, whose egg size aligns with lactation, *Lystrosaurus* anchors the plesiomorphic condition deep within Synapsida. Its reproductive strategy may have played a crucial role in its resilience and ecological dominance following the end-Permian mass extinction.

## Introduction

The origin of the amniotic egg is considered a landmark in vertebrate evolution [1–3]. Based on the reproductive biology of modern monotremes, oviparity (egg-laying) is consensually considered the ancestral condition for Synapsida, the ancient evolutionary line of amniotes that leads to mammals [1,3,4]. The eggshell of early amniotes was likely soft [5,6], and consequently, the fossil record of late Palaeozoic and early Mesozoic amniotic eggs is patchy and contested. The oldest possible amniotic egg belongs to a mesosaurid sauropsid from the early Permian of South America [7]. The shell is not preserved, but the small size, skeletal immaturity, and curled-up position of the embryo strongly support that it was *in ovo*. The oldest confirmed amniotic eggs preserved with their shell and embryo belong to sauropodomorph dinosaurs from the Early Jurassic of Gondwana [5,8–11]. The oldest possible fossil amniotic egg attributed to synapsids (based on the presence of copious pelycosaur remains from the same horizon) is from the early Permian of North America [1,2,12], but in the absence of an embryo or convincing shell-like crystalline microscopic structure, this identification is no longer supported [13].

In the South African Karoo Basin, the Elliot Formation preserves embryonated dinosaur eggs alongside skeletal remains of non-mammalian cynodonts [9,14,15]. Despite these favourable premises, no convincing synapsid egg has ever been discovered. As early as 1964, renowned South African palaeontologist James Kitching expressed concern over this issue, even going so far as to question whether Permo-Triassic synapsids laid eggs at all [16,17]. Considering i) the extreme abundance of some therapsid taxa, such as *Lystrosaurus* and *Diictodon* [14,18], ii) that synapsid perinates have been found in the Karoo Basin and elsewhere [14,16,19–21], iii) the apparent absence of bias against egg preservation in Karoo rocks [5,10,14], and finally, iv) the 180 years of continuous palaeontological efforts since the description of the first early synapsid [22,23], the persisting lack of any convincing Palaeozoic or Mesozoic synapsid fossil egg remains puzzling and difficult to explain [3,24]. The frequency and relative ease with which modern sauropsids can shift between oviparity, ovoviviparity, and viviparity [25–27] and the results of recent phylogenetic approaches to the question of egg evolution in amniotes [28] echo Kitching’s reservations about whether egg-laying was the ancestral reproductive biology of synapsids. Thus, although egg-laying is currently widely accepted as the ancestral reproductive strategy for Permo-Triassic synapsids, the supporting evidence remains, at best, circumstantial [21,24].

This lack of evidence has rippling effects beyond the field of synapsid palaeontology. For instance, the foundations of the currently accepted evolutionary hypotheses to account for the origin of lactation in mammals rely heavily on an early egg-laying stage [29–34]. It is generally agreed that milk did not initially evolve for feeding, but as skin secretions used to either moisturise the eggs, provide nutrients, protect them against fungi and bacterial infections, or for hormonal signalling through the egg membrane [31–36]. This hypothesis would collapse if non-mammalian synapsids were not egg-laying and the monotreme oviparity was unique (i.e., autapomorphic). This exemplifies the crucial importance of robust and evidence-based reconstructions of the ancestral reproductive biology in the synapsid lineage.

**Fig 1:**
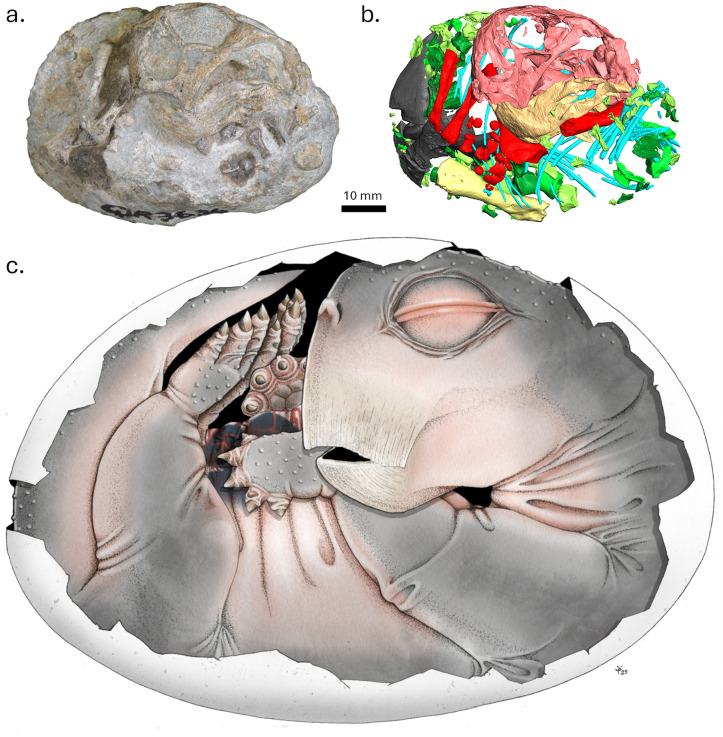
Specimen NMQR 3636 in left lateral view. a, photograph of the specimen; b, 3D digital reconstruction of the segmented bones; c, live reconstruction by artist Sophie Vrard. Colour code for b: vertebral elements in shades of green, ribs in blue, forelimb elements in red, femur in yellow, pelvic girdle elements in grey, skull in light red, mandible in light orange.

We used X-ray micro-computed tomography (CT) scanning and synchrotron radiation X-ray (SRCT) imaging to study the three smallest known, perinate specimens of the early Triassic (Induan) dicynodont synapsid *Lystrosaurus*. One of the specimens is preserved in a curled posture and shows tangible evidence that it was preserved *in ovo* (Fig 1), thus demonstrating oviparity for the first time in early synapsids.

## Materials and methods

**Fig 2:**
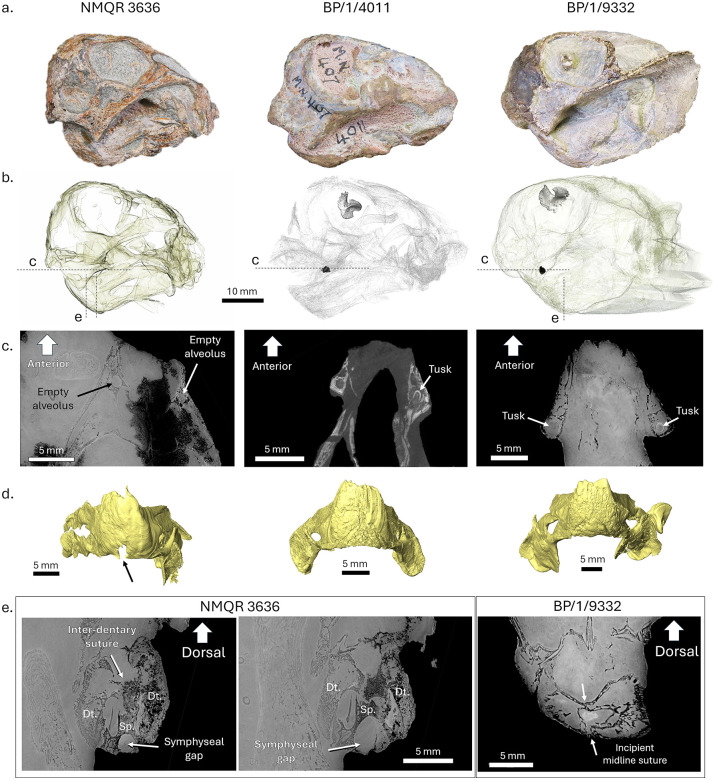
Comparison of cranial features of perinate *Lystrosaurus* specimens. From left to right, NMQR 3636, BP/1/4011, and BP/1/9332. a, photographs of the skulls in lateral view; b, 3D digital transparent skull showing the tusk (in black) and mesethmoid (in grey), if preserved. The dotted lines marked “c” and “e” indicate the planes of view displayed in the sections of panel c and e; c, coronal CT section through the tusk sockets; d, 3D model of lower jaws in anterior view to display the symphysis. The black arrow points to the symphyseal gap in NMQR 3636; e, CT cross sections through the mandibular symphysis in two positions in NMQR 3636 (left and middle) and BP/1/9332 (right) showing the state of ossification of the rostral mandibular bones. Arrows point to incompletely co-ossified sutures. Abbreviations: Dt., dentary; Sp., splenial.

The specimens studied here are the three smallest known *Lystrosaurus*, BP/1/9332, BP/1/4011, and NMQR 3636, with skull lengths of 44.0 mm, 43.0 mm and 34.5 mm, respectively (Fig 2a). They all come from the Induan (Early Triassic) of South Africa (following the most generally accepted dates [37,38], but see [39]). All necessary permits were obtained for the described study, which complied with all relevant regulations. The specimens were collected and studied under the South African Heritage Resources Agency permits 4214 (CaseID: 23376) and 4118 (CaseID: 19929).

NMQR 3636: Almost complete curled up skeleton of an early juvenile *Lystrosaurus* sp., with affinities to *L. murrayi*. Provenance: farm Rheeboksfontein 5 (alternate spellings: Reboksfontein, Rheboksfontein) (Xhariep Municipal District, Free State Province, South Africa). Found by John Nyaphuli in 2008. No GPS coordinates available. Stratigraphy: the 1:250 000 geological map of the area (Council for Geoscience, 1997) suggests this locality lies on both latest Permian and Early Triassic rocks, but our recent fieldwork in the area [40] indicates that the outcrops here belong to the upper Palingkloof Member of the Balfour Formation or the lower Katberg Formation. This gives an Induan age for this fossil. The specimen was imaged at the BM18 beamline of the European Synchrotron Radiation Facility (ESRF), Grenoble, France, with a voxel size of 17.27 µm (see S1 Data; [41]).

BP/1/4011: Isolated skull, currently, the smallest *Lystrosaurus* skull that has been figured. It was briefly described as the smallest known *Lystrosaurus* specimen by Kitching [16] and Grine et al. [42] as BP MN 407. It was formerly identified as *L. murrayi* or *L. curvatus* [42], but is here more cautiously considered as a *Lystrosaurus* sp., with affinities to *L. curvatus*. Found by James Kitching. No GPS coordinates available. Provenance: “Orangia” on Tweefontein 508 (Xhariep Municipal District, Free State Province, South Africa). Stratigraphy: upper Palingkloof Member of the Balfour Formation, early Induan [43]. Scanned at the ESI using Nikon Metrology XTH 225/320 LC with voxel size: 0.0393 mm.

BP/1/9332: Almost complete and articulated skeleton of an early juvenile of *Lystrosaurus* sp., with affinities to *L. murrayi*. Locality: farm Nooitgedacht 68 (Spitskop), Xhariep Municipal District (previously Bethulie District, Free State Province, South Africa). Found by Brandon Stuart. GPS: S 30° 20’ 12.8” E 25° 55’ 41.5” Stratigraphy: upper Palingkloof Member of the Balfour Formation, early Induan [44]. The specimen was imaged at the BM18 beamline of the ESRF, Grenoble, France, with a voxel size of 6.62 µm (see S1 Data; [41]).

The specimens were studied digitally using manual segmentation in Avizo 2021 (FEI, Hillsboro, OR, USA). 3D digital volumes of all three specimens are available as S2–S4 Data. Egg volume was reconstructed based on linear measurements (maximum length and width) of NMQR 3636 and the formula to calculate the volume of an ellipsoid:


$${\text{V}}_{\text{egg}}\;=\;\text{4}/\text{3}\;\times \;\pi \;\times \;\text{3}.\text{65 cm }\times \;\text{2}.\text{75 cm }\times \;\text{2}.\text{75 cm}$$

The resulting volume, 115 cm³, exceeds that of the actual fossil, which is expected given that i) the skeleton is incomplete and compressed (see description below), and that ii) the egg would also have included an unknown quantity of yolk. Despite this, the length and width of the fossil can be safely interpreted as accurately reflecting the dimensions of the egg because the skeleton is tightly packed inside the space delineated by the vertebral column, which curls along what was likely the interior of the soft eggshell (Fig.1). Thus, despite the volume difference noted above, the current estimate is, in fact, a conservative estimation of the volume of the egg. This gives an egg mass of approximately 115 g, assuming a density equal to that of water.

The egg volume of *Lystrosaurus* was compared to those of other amniotes using the dataset of Werner and Griebeler [45] (S5 Data). No osteohistologically mature Triassic *Lystrosaurus* is currently known in Southern Africa [46,47] as high juvenile mortality was prevalent during the end-Permian mass extinction (EPME) and individuals demonstrably died at young ontogenetic stages [48,49]. Given this, we used three different estimates of body mass for Triassic *Lystrosaurus* currently available in the literature: i.e., 8.825 kg, 18.511 kg, and 50.000 kg [50–52]. For monotremes, egg volumes and body mass estimates are from Griffiths’ and Macrini’s works, respectively [4,53]. Body mass and egg volume for the tritylodontid cynodont *Kayentatherium* are from Hoffman and Rowe [21].

### Institutional abbreviations

BP, Evolutionary Studies Institute (formerly, Bernard Price Institute) of the University of the Witwatersrand (Johannesburg, South Africa); BP MN, Bernard Price Museum Number; NMQR, National Museum (Bloemfontein, South Africa).

## Results

### Comparison between juveniles

Cluver [54], Grine et al. [42], Botha et al. [48], and Botha [46] have already provided comprehensive studies on the skeleton and ontogeny of *Lystrosaurus*. This contribution thus focuses on the new anatomical data obtained from comparing BP/1/9332, BP/1/4011, and NMQR 3636.

Based on their basal skull length (length from the basioccipital condyle to the anterior edge of the premaxilla), these specimens are the three smallest *Lystrosaurus* ever found [42]. Specimen NMQR 3636 is the smallest, with a basal skull length of 34.5 mm. The skull of BP/1/4011 is 43.0 mm long and that of BP/1/9332 is 44.0 mm long. None of the specimens preserves an egg tooth (caruncle). An egg tooth is normally present in neonate monotremes [4,55], thus it is possible that, here, it was lost, not preserved, or damaged during preparation. Both BP/1/4011 and BP/1/9332 possess small unerupted tusk buds visible in their maxillary alveolae, whereas NMQR 3636 has empty alveolae (Fig 2b, c). The mesethmoid bone, which supports the olfactory bulbs in life, is completely ossified in both BP/1/4011 and BP/1/9332. It is displaced in the former, whereas it is preserved in situ in the latter, suggesting that the median septal cartilage that supported it was better developed in BP/1/9332. Specimen NMQR 3636 preserves no mesethmoid. It may have been lost or completely cartilaginous and therefore not preserved. The occipital and basicranial bones, including the supraoccipital, tabulars, prootics, opisthotics, exoccipitals, and basioccipital, are loose and displaced in both NMQR 3636 and BP/1/4011. As a result, the occipital surface of the skull of these two specimens has been crushed, which gives it a posteriorly tapering profile in lateral view (Fig 2a). In contrast, the occipital bones are preserved in situ and in articulation in BP/1/9332, and its skull is undeformed as a result (Fig 2a). In *Lystrosaurus*, the co-ossification of cranial bones begins anteriorly and proceeds posteriorly, so that the occipital bones are the last to co-ossify [54]. This suggests that the development of BP/1/9332 was more advanced than that of the other two. Sclerotic ring plates are preserved in all three specimens, demonstrating that these bones formed very early in ontogeny.

The lower jaw in NMQR 3636 displays an unfinished intermandibular symphysis. Ventrally, on the mandible, a deep notch excavates the splenials at the midline, showing that the symphyseal suture between the two paired bones is incompletely co-ossified (Fig 2d). The edges of this notch are smooth on synchrotron images, which demonstrates that this feature is not due to post-mortem damage or erosion (Fig 2e). It likely accommodated Meckel’s cartilage [56]. Dorsally, the inter-dentary suture is still clearly open in NMQR 3636 (Fig 2e). In contrast, BP/1/4011 and BP/1/9332 reveal splenials that are completely co-ossified and the inter-dentary suture is mostly closed, although a midline suture is still visible at mid-height on the symphysis (Fig 2d, e).

**Fig 3:**
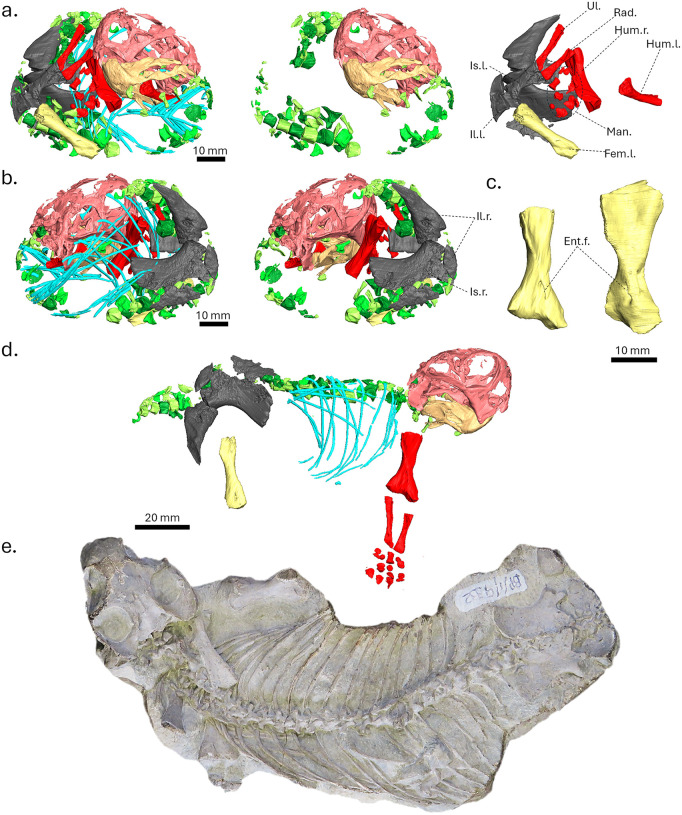
Comparison of two perinate *Lystrosaurus* skeletons. a, 3D digital reconstruction of NMQR 3636 in left lateral view showing the complete skeleton (left), the skull, lower jaw and vertebrae only (middle), and the pelvis and femur only (right); b, 3D digital reconstruction of NMQR 3636 in right lateral view with (left) and without the ribs (right); c, comparison between the right humerus of NMQR 3636 and the left humerus (mirrored) of BP/1/9332; d, reconstructed lateral view of the preserved skeletal elements belonging to NMQR 3636; e, photograph of BP/1/9332 in dorsal view. Colour code for a and b: vertebral elements in shades of green, ribs in blue, forelimb elements in red, femur in yellow, pelvic girdle elements in grey, skull in light red, mandible in light orange. Abbreviations: Ent.f., entepicondylar foramen; Fem., femur; Hum.l., left humerus; Hum.r., right humerus; Il.l., left ilium; Il.r., right ilium; Is.l., left ischium; Is.r., right ischium; Man., manus; Rad., radius; Ul., ulna.

Two of the three specimens preserve postcrania, i.e., BP/1/9332 and NMQR 3636. Since neither specimen has duplicate elements, each likely represents a single individual. Specimen NMQR 3636 is preserved in a curled-up position in a 73.0 mm long and 5.5 mm wide nodule. It preserves most of the vertebral column up to the last caudal vertebra, the left humerus, most of the right forelimb, including right humerus, radius, ulna and undetermined and disarticulated elements of the right manus, as well as most of the pelvis and the left femur (Fig 3a, b, d). The carpals are spherical due to the incomplete ossification of the hand. Both the humerus and femur have unossified epiphyses (Fig 3a-c). On the vertebrae, the centra and neural arch are separated on all vertebrae, and many vertebral elements are loose and displaced. In the pelvic region, the sacral vertebrae and connecting ribs are loose and disarticulated, resulting in the absence of a co-ossified sacrum. A few of the left and right dorsal ribs are preserved and are tightly packed around the skeleton.

Specimen BP/1/9332 is preserved in a splayed-out position (Fig 3e), typical of most *Lystrosaurus* articulated skeletons found in the Karoo Basin [49]. All bones of the skeleton are almost perfectly articulated, as synchrotron images show that no loose elements are preserved in the surrounding matrix. The most distal elements were lost to erosion. The forelimbs preserve both humeri and the proximal half of the left radius and ulna. Both hind limbs are missing except for a small fragment of the right femur (Fig 3e). The epiphyses of the humeri demonstrate a more advanced degree of ossification than in NMQR 3636 (Fig 3c). As in NMQR 3636, the centra and neural arches of the vertebrae are unfused. The pelvis is preserved in full anatomical articulation, which suggests a more advanced stage of development of the cartilages in this specimen compared to NMQR 3636 (Fig 3e). The sacrum includes six sacral vertebrae with ribs connecting them to the pelvic bones.

Osteohistological differences are minor between all three individuals (S6 Data). The bone microstructure is mostly spongy. No hatchling line is visible in the long bones of NMQR 3636 and BP/1/9332, consistent with previous observations made in juvenile *Lystrosaurus* specimens [46,47,57].

**Fig 4:**
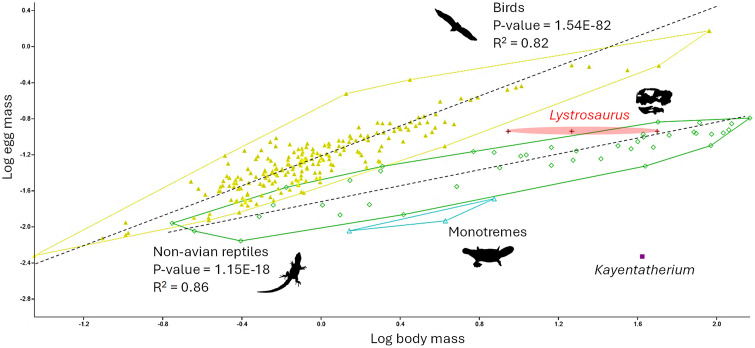
Plot of egg mass against body mass in amniotes (logged). Colour code: red, *Lystrosaurus*; Purple, *Kayentatherium*; Yellow, birds; Green, non-avian reptiles. Data after [45].

Working on the hypothesis that NMQR 3636 is the skeleton of a *Lystrosaurus* preserved in its egg (see discussion below), its estimated volume would be 115 cm^3^ and its mass would be 115 g (assuming a density equal to that of water). Given the range of body masses considered here for Triassic *Lystrosaurus* (see material and methods), its estimated egg sizes are largely consistent with other egg-laying amniotes (Fig 4). They fall in the upper range of those of reptiles for the largest body mass considered, while they fall slightly above them for smaller body masses. Monotremes have comparatively smaller eggs than other amniotes because of the limited amount of yolk they contain, which is compensated for by post-hatching milk feeding [4,32]. The values for *Kayentatherium* [21] make it fall far outside other amniotes on the scatter plot, well below the values for reptiles and monotremes (Fig 4).

## Discussion

### Identification of NMQR 3636 as an *in ovo* embryo

All three specimens have similar skull lengths (approximately 4 cm) and were thus almost the same age at the time of death, likely perinates given their very small size and low level of skeletal ossification. They can be organised in a growth sequence, beginning with NMQR 3636, which is likely at the youngest developmental stage, owing to its incomplete mandibular symphysis, absence of tusk buds, possible absence of ossified mesethmoid, and lesser degree of occipital bone co-ossification. Specimen NMQR 3636 also has the shortest skull. Specimen BP/1/4011 is the second least mature of the three specimens because its splenials are co-ossified at the mandibular symphysis (unlike in NMQR 3636), but its occipital and basicranial bones remain loose (unlike BP/1/9332). Its skull length is intermediate between the other two specimens. Skeleton BP/1/9332 is the most developed of the three, as it has the longest skull and its occipital bones are sutured together. Its humeral epiphyses are also in a more advanced state of ossification than those of NMQR 3636. Its splayed-out position shows it is not preserved in an egg and had moved around for some distance before dying (Fig 3e).

Specimen NMQR 3636 was therefore likely younger at the time of death than the other two specimens, making it the least developed of all known *Lystrosaurus* skeletons. The evidence that supports NMQR 3636 was *in ovo* when it died include the dimensions of the nodule in which it is preserved, which are consistent with that of a non-avian amniote egg (Fig 4), and the curled-up position of the skeleton that outlines an ovoid shape consistent with that of an egg (Fig 1). Elsewhere, finding a perinate individual in this position would be sufficient to reasonably hypothesise *in ovo* preservation in other fossil amniotes, even in the absence of a preserved egg shell (e.g., [7,28]); however, in the Permo-Triassic Main Karoo Basin, many early juvenile, adult, and subadult tetrapods have been found fossilised in this position (e.g., [15,20,58,59]). Thus, the curled-up position alone is not enough to conclude that NMQR 3636 was *in ovo*.

The disarticulation of the pelvis, sacral vertebrae, and ribs suggests that the pelvic bones and cartilage were too weak to support its weight, unlike those of the splayed-out and more mature BP/1/9332. This is consistent with an *in ovo* occurrence. Furthermore, in NMQR 3636, there is a large gap on the ventral side of the lower jaw where the two splenial bones are not fully co-ossified medially. The edges of this gap are smooth and undamaged, indicating that it is a genuine anatomical feature (Fig 2c, d), that likely accommodated Meckel’s cartilage in life. In other tetrapods with a beak, i.e., chelonians and birds, the symphysis of the lower jaw always closes *in ovo*, in the last third of embryogenesis [60–65]. Synchrotron images of *in ovo* embryos of the turtle *Centrochelys sulcata* show that the mandibular symphysis becomes fully co-ossified between 45 and 55 days of embryogenesis [66–68]. This supports that NMQR 3636 was not yet close to the hatching stage. Egg-laying mammals have unfused mandibles when they are born, but they are highly altricial because they are fed milk before they complete their development [4], whereas *Lystrosaurus* most likely did not produce milk [32,69]. It is unlikely that a *Lystrosaurus* hatchling with an unfused, partly cartilaginous intermandibular symphysis would have been able to feed itself and survive, as its jaws would have been too weak to process hard food items. Altogether, all the evidence supports the conclusion that NMQR 3636 is preserved in a curled-up, *in ovo* position.

### Egg size and the reproductive biology of *Lystrosaurus*

Comparing egg sizes amongst amniotes reveals that *Lystrosaurus* laid relatively large eggs for a non-avian amniote, regardless of the body size considered (Fig 4). This is remarkable as the egg size reconstructed and compared here is a minimum value based on an incompletely developed and partly damaged embryo. Thus, the following conclusions are based on a conservative estimate of Triassic *Lystrosaurus* egg size.

In extant tetrapods, larger eggs generally correlate with precociality and higher levels of parental investment in raising the hatchling [70,71]. Accordingly, the relatively large size of the egg in Triassic *Lystrosaurus* compared to other egg-laying, terrestrial amniotes (Fig 4) aligns with the rich fossil record of adult-juvenile aggregations of late Palaeozoic and early Mesozoic synapsids that has been traditionally interpreted as illustrating the early evolution of parental care in this lineage [19–21,52,72–74]. Large egg size also correlates with low predation and low competition in extant species [71,75], which is consistent with the depleted terrestrial biodiversity after the EPME, in which early Triassic *Lystrosaurus* were the only medium-sized herbivorous species, and predators large enough to attack them (e.g., *Moschorhinus*, *Proterosuchus*) were scarce [37]. Larger eggs are also less prone to desiccation, particularly soft-shelled ones, because their surface area to volume ratio is low [76,77]. This would have been advantageous in the arid post-extinction environment [49,78].

*Lystrosaurus* early juveniles or neonates are usually discovered as single specimens, but rare groups of two or three individuals arguably belonging to the same age class have also been found [14,16,79,80]. The only exception is an aggregation of nine more mature juvenile *Lystrosaurus* specimens, which are disarticulated and thus likely gathered post-mortem [49,79]. This would imply a rather small to medium-sized clutch, which, combined with the relatively large size of the eggs, would be consistent with precociality [11], as is indicated by the early mobility of the neonate individual BP/1/9332 hypothesised above. In addition, this specimen and BP/1/4011 both have closed mandibular symphyses with only an incipient suture visible (Fig 2d) indicating that they would have been able to process hard food items. Larger eggs and precociality would have been advantageous in the post-EPME environment as they would have enhanced the exploitation of the scarce food resources soon after hatching [71]. Precociality would also be consistent with Triassic *Lystrosaurus* reaching sexual maturity early, a trait favoured by natural selection as juvenile mortality was very high [46–48].

Neonates of the genus *Diictodon*, a dicynodont taxon from the Permian that survived the end-Capitanian extinction, are also found as single individuals or in pairs [19], whereas known aggregations of juveniles of other dicynodont species are significantly larger [74,81,82]. This may be because small clutch size could have been a recurring survival strategy during biological crises in this clade. Alternatively, large aggregations of dicynodonts (including *Lystrosaurus*) often involve more mature juveniles and/or subadults [74,79,82], thus it is possible that social dicynodonts were gathering in increasingly larger groups as they became older. More data is needed to address these possibilities.

### Implications for the evolution of lactation

In extant egg-laying mammals— monotremes—the limited yolk supply is compensated for by lactation. As a result, their eggs are comparatively small (Fig 4), and the hatchlings are highly altricial [4,30,33]. Consequently, the reconstructed range of egg size presented here rules out the possibility that *Lystrosaurus* produced milk for feeding (Fig 4). In contrast, the egg size to body mass ratio of the non-mammalian tritylodontid cynodont *Kayentatherium* is very low (Fig 4). Given its large clutch size (38 individuals based on a specimen from the Early Jurassic), it has been proposed that *Kayentatherium* had an essentially reptilian-like reproductive biology [21]. This, coupled with evidence of dental wear in neonates, indicated that hatchlings were partially capable of processing hard food items and that adult female *Kayentatherium* likely did not lactate [24]. The current results nuance this interpretation, as the very small size of the neonates in the aggregation suggests that *Kayentatherium* had very small eggs compared to *Lystrosaurus* and other amniotes (Fig 4), more consistent with the size of the eggs in monotremes [4]. Dental wear at birth is found in some extant rodents, which shows that this trait is not incompatible with lactation [83]. Moreover, as proposed by Hopson [29], it is probable that primitive milk was provided alongside other food items to sustain the young in the first evolutionary steps towards mammalian lactation. Under these circumstances, dental wear in neonate tritylodontid cynodonts is not unexpected. The evolution of primitive milk production at the evolutionary root of the Mammaliamorpha, including tritylodontids, would be consistent with the timing of some mutations involving genes coding for caseins and vitellogenin proteins (for the production of milk and reduction of egg yolk, respectively), and MSX2, a gene involved in the formation of mammary glands [34,69,84–86]. Tritylodontids also likely had hair [87–89], which further supports that they had mammary glands, given the well-established genetic, phylogenetic, morpho-anatomical, and ontogenetic connections between the two traits [32,34,69]. Thus, the previous observations made on *Kayentatherium* are, here, reconciled with a possibly more mammal-like reproductive biology than previously envisioned, including lactation. They, in fact, support that *Kayentatherium* was indeed more mammal-like in this respect than the more basal *Lystrosaurus*.

## Conclusion

The shape, size, curled-up posture, weak limb and pelvic ossification, and unfused lower jaw symphysis of the *Lystrosaurus* specimen NMQR 3636 all support the interpretation that it represents an *in ovo* individual. The large egg size and its skeletal features are consistent with a non-milk-feeding, precocial animal, providing crucial data for reconstructing the evolutionary origins of lactation at the root of the mammalian evolutionary tree. As a non-cynodont synapsid and survivor of the EPME, *Lystrosaurus* occupies a pivotal position for understanding how reproductive strategies shaped survival during this extinction. Modern mammals—including monotremes, marsupials, and placentals—exhibit a wide range of reproductive strategies, and in this context, NMQR 3636, along with emerging insights from the more mammalian tritylodontid cynodont *Kayentatherium*, offers a valuable anchor point for determining the polarity and sequence of key reproductive traits in early mammalian evolution. This exceptional *Lystrosaurus* fossil not only informs our understanding of developmental biology in non-mammalian synapsids but also sheds light on the adaptive strategies that may have contributed to their resilience in the face of mass extinction.

## Supporting information

S1 Data Scanning parameters of NMQR 3636 and BP/1/9332. (DOCX)

S2 Data STL files of NMQR 3636. (ZIP)

S3 Data STL files of BP/1/9332. (ZIP)

S4 Data STL files of BP/1/4011. (ZIP)

S5 Data Dataset of egg and body mass in tetrapods (modified from [45]). (XLSX)

S6 Data Supplementary Figure 1. (DOCX)
