# Supplementary material for: The first non-mammalian synapsid embryo from the Triassic of South Africa
Source: PLoS One. 2026 Apr 9;21(4):e0345016. doi: 10.1371/journal.pone.0345016 (PMC13065020; doi:10.1371/journal.pone.0345016)
Supplement: S1 Data — (DOCX) [file pone.0345016.s001.docx]

**Supplementary Data 1**

**Propagation phase contrast synchrotron X-ray micro-computed tomography**

Specimens BP/1/9332 and NMQR 3636 were imaged at the BM18 beamline of the European Synchrotron Radiation Facility, Grenoble, France, using propagation phase contrast synchrotron X-ray micro-computed tomography. Both acquisitions were performed in filtered white beam, in helical geometry (see Supplementary Data Table 1, below, for acquisition parameters, and Benoit et al. 2025). Tomographic reconstructions were done with a derived version of the Nabu software (Paleo et al. 2019), using a single distance phase retrieval approach (Paganin et al., 2002, Paganin et al. 2020) resulting in 32-bits tiffs stack. Post processing included: change of the dynamic range to 16-bits, discarding 32-bits values outside the 0.001% minimum and 99.999% maximum percentiles; ring correction on slices (Lyckegaard et al., 2011); cropping of the data; and generation of a binning 2x2x2 duplicate of the datasets to facilitate data analysis. Matlab code used for the post-processing are available <https://github.com/HiPCTProject/Tomo_Recon>

Table 1 - List of parameters for propagation phase contrast synchrotron X-ray micro-computed tomography acquisition on the BM18 beamline of the ESRF

| **Scan Name** | HA2000_17.27um_BP-1-9332_Lystrosaurus_complete | HA2200_6.52um_QR-3636_Lystrosaurus |
| --- | --- | --- |
| **beamline** | bm18 | bm18 |
| **SR Current (mA)** | 199.134 | 199.517 |
| **Filters** | sapphire 5mm, lead brass 10mm rods 2x5mm | Mo 3.75mm |
| **Energy (keV)** | 186 | 150 |
| **sample-detector-distance (mm)** | 20000 | 6000 |
| **Camera** | IRIS_15 | IRIS_15 |
| **magnification** | 0.125x | 0.589x |
| **Pix size (µm)** | 17.271 | 6.523 |
| **proj size (X)** | 5056 | 5056 |
| **proj size (Y)** | 600 | 1538 |
| **binning** | 1x1 | 1x1 |
| **Scintillator** | LuAG2000 with reflective layer | LuAG2000 |
| **Sequence** | tomo:helical | tomo:helical |
| **sz-start (mm)** | -248.72 | -337 |
| **sz-end (mm)** | -46.302 | -272.976 |
| **length (mm)** | 202.418 | 64.024 |
| **sz step (mm)** | 4.307 | 3.766 |
| **overlap** | 41.6% | 37.5% |
| **total number of projections** | 14224 | 14852 |
| **COR motor yrot (mm)** | 34.542 | 14.351 |
| **COR Offset (px)** | 2000 | 2200 |
| **Exposure subframe (s)** | 0.01 | 0.025 |
| **accumulation** | 3 | 3 |
| **Exposure total (s)** | 0.03 | 0.075 |
| **Dark images N** | 200 | 100 |
| **Flatfield images N** | 201 | 101 |
| **Dark average (adu)** | 301 | 306 |
| **flatfield aveerage (adu)** | 40512 | 36716 |

**Additional references**

Benoit, J., Lund, E., & Fernandez, V. (2025). Propagation phase contrast synchrotron X-ray micro-computed tomography datasets of two fossil of Lystrosaurus, BP-1-9332, and NMQR 3636 (Version 1) [Dataset]. European Synchrotron Radiation Facility. doi.org/10.15151/ESRF-DC-2272014371

Lyckegaard A., Johnson G.& Tafforeau P. (2011). Correction of ring artifacts in X-ray tomographic images. *Int. J. Tomo. Stat.* 18, 1-9.

Paganin D., Mayo S., Gureyev T.E., Miller P.R.& Wilkins S.W. (2002). Simultaneous phase and amplitude extraction from a single defocused image of a homogeneous object. *Journal of microscopy.* 206, 33-40.

Paganin, D.M., Favre-Nicolin, V., Mirone, A., Rack, A., Villanova, J., Olbinado, M.P., Fernandez, V., da Silva, J.C. and Pelliccia, D., 2020. Boosting spatial resolution by incorporating periodic boundary conditions into single-distance hard-x-ray phase retrieval. *Journal of Optics*, *22*(11), p.115607.

Paleo P., Mirone A., Nemoz C. and Viganò N. R.,  2019,  Nabu. https://gitlab.esrf.fr/tomotools/nabu
