# Supplementary material for: The first non-mammalian synapsid embryo from the Triassic of South Africa
Source: PLoS One. 2026 Apr 9;21(4):e0345016. doi: 10.1371/journal.pone.0345016 (PMC13065020; doi:10.1371/journal.pone.0345016)
Supplement: S6 Data — (DOCX) [file pone.0345016.s006.docx]

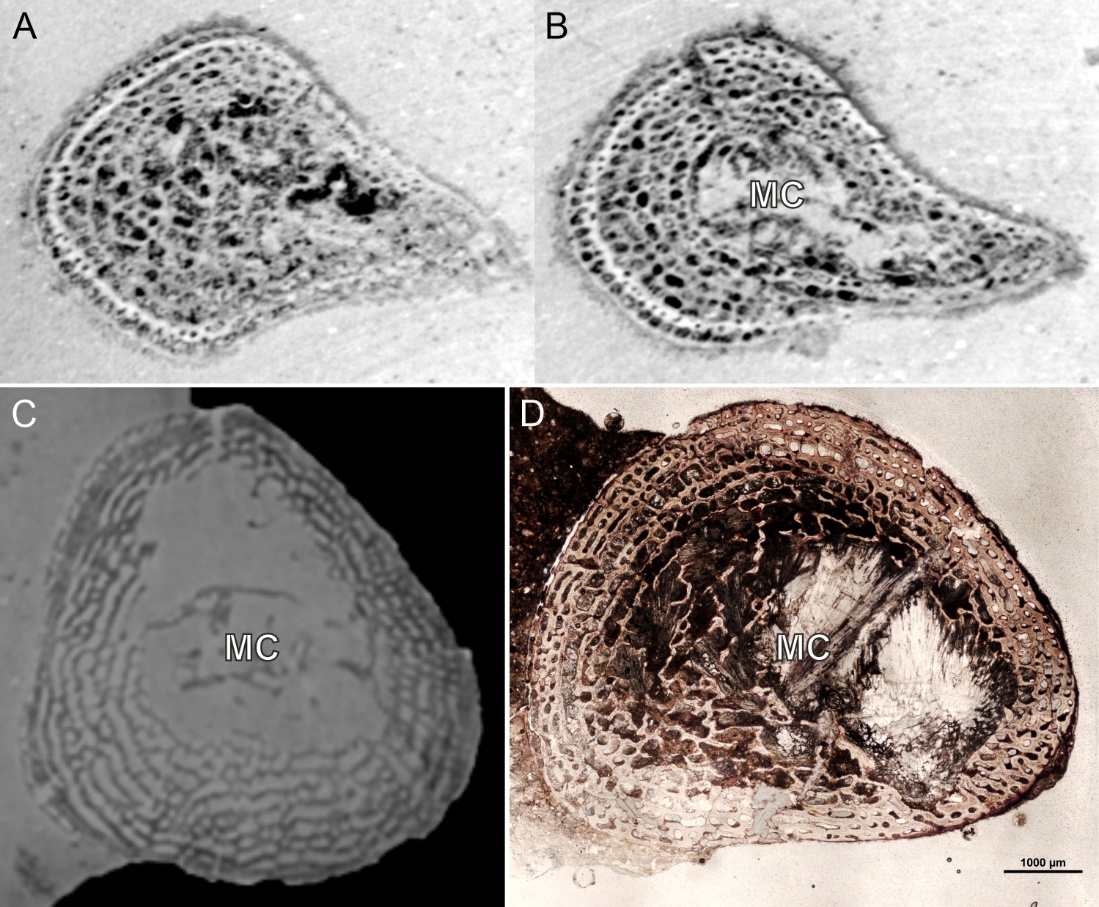


**Supplementary Figure 1.** Humeral osteohistology of *Lystrosaurus*. A, NMQR 3636 closer to the midshaft; B, NMQR 3636 further into the deltopectoral crest region. Both images reveal a spongy cortex with specular bone and an unclear medullary cavity. C, BP/1/9332 in the midshaft showing spongy bone with more circumferential canals; D, SAM-PK-3531 in the midshaft showing a woven-parallel complex and a clear medullary cavity. Abbreviations: MC, medullary cavity.

**Description and comparison**

The bone microstructure of the humerus of NMQR 3636 reveals a typical spongy bony scaffold typical of very young vertebrates (Supp. Fig. 1A, B). Although the resolution is not high enough to detect osteocyte lacunae, it is well known from previous research on *Lystrosaurus* [e.g. 44] that very small, presumably young individuals exhibit a woven bone scaffold. The humerus of the larger BP/1/9332 reveals similar spongy bone (Supp. Fig. 1C) and a slightly larger individual (humerus of Sam-pk-3531, basal skull length 49 mm, [44]) reveals highly vascularized woven bone and primary osteons (i.e. woven-parallel complex) (Supp. Fig. 1D). Slight differences between NMQR 3636 and the larger individuals include a clearer medullary cavity and a higher incidence of circumferential vascular canals in the latter. NMQR 3636 is dominated by more longitudinally oriented canals and has a slightly more specular appearance (less bone), but these differences are minor as the osteohistology of all three individuals is relatively similar. No hatchling line was detected in NMQR 3636, however, such a line has not been noted for any *Lystrosaurus* specimens to date [1, 2; 3].

**Reference list**

1. Botha, J. The paleobiology and paleoecology of South African *Lystrosaurus*. *PeerJ* **8**, e10408 (2020).

2. Kulik, Z. T., Lungmus, J. K., Angielczyk, K. D. & Sidor, C. A. Living fast in the Triassic: New data on life history in *Lystrosaurus* (Therapsida: Dicynodontia) from northeastern Pangea. *PLOS ONE* **16**, e0259369 (2021).

3. Han, F., Zhao, Q. & Liu, J. Preliminary bone histological analysis of *Lystrosaurus* (Therapsida: Dicynodontia) from the Lower Triassic of North China, and its implication for lifestyle and environments after the end-Permian extinction. *PLOS ONE* **16(3)**:e0248681 (2021).
